# 6-Phenyl-5a,6,6a,7,12,13a-hexa­hydro-5*H*-benzo[6,7]indolizino[3,2-*a*]pyrrolizine

**DOI:** 10.1107/S1600536809020091

**Published:** 2009-06-06

**Authors:** B. Gunasekaran, S. Kathiravan, R. Raghunathan, V. Renuga, V. Manivannan

**Affiliations:** a Department of Physics, AMET University, Kanathur, Chennai 603 112, India; b Department of Organic Chemistry, University of Madras, Guindy Campus, Chennai 600 025, India; c Department of Chemistry, National College, Thiruchirapalli, Tamil Nadu, India; d Department of Research and Development, PRIST University, Vallam, Thanjavur 613 403, Tamil Nadu, India

## Abstract

In the title compound, C_23_H_22_N_2_, the central pyrrolidine ring adopts an envelope conformation. The benzene ring of the hexa­hydro­pyrroloisoquinoline ring system makes dihedral angles of 83.43 (6) and 61.99 (10)°, respectively, with the phenyl and pyrrole rings. In the crystal structure, weak C—H⋯π inter­actions are observed.

## Related literature

For biological activity of pyrrolidine derivatives, see: Witherup *et al.* (1995 ▶); Kravchenko *et al.* (2005 ▶). For biological activity of pyrrole derivatives, see: Sobral & Rocha Gonsalves (2001*a* ▶,*b* ▶); Brockmann & Tour (1995 ▶); Suslick *et al.* (1992 ▶); Di Natale *et al.* (1998 ▶). For a related structure, see: Liu *et al.* (2007 ▶).
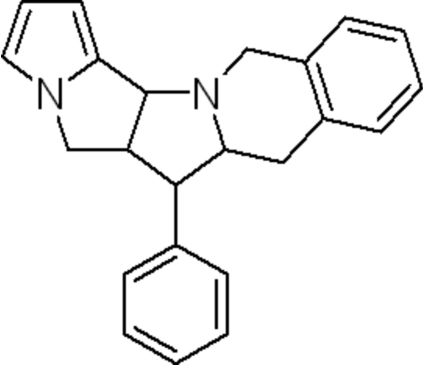


## Experimental

### 

#### Crystal data

C_23_H_22_N_2_
*M* *_r_* = 326.43
Monoclinic, 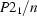

*a* = 14.0694 (14) Å
*b* = 5.9300 (5) Å
*c* = 21.177 (2) Å
β = 104.563 (3)°
*V* = 1710.1 (3) Å^3^
*Z* = 4
Mo *K*α radiation
μ = 0.07 mm^−1^
*T* = 293 K
0.20 × 0.20 × 0.15 mm

#### Data collection

Bruker Kappa APEXII diffractometer
Absorption correction: multi-scan (**SADABS**; Sheldrick, 1996 ▶) *T*_min_ = 0.985, *T*_max_ = 0.989
20373 measured reflections
4266 independent reflections
3049 reflections with *I* > 2σ(*I*)
*R*_int_ = 0.032

#### Refinement

*R*[*F* ^2^ > 2σ(*F* ^2^)] = 0.049
*wR*(*F* ^2^) = 0.149
*S* = 1.01
4266 reflections
226 parameters
H-atom parameters constrained
Δρ_max_ = 0.17 e Å^−3^
Δρ_min_ = −0.23 e Å^−3^

### 

Data collection: *APEX2* (Bruker, 2004 ▶); cell refinement: *SAINT* (Bruker, 2004 ▶); data reduction: *SAINT*; program(s) used to solve structure: *SHELXS97* (Sheldrick, 2008 ▶); program(s) used to refine structure: *SHELXL97* (Sheldrick, 2008 ▶); molecular graphics: *PLATON* (Spek, 2009 ▶); software used to prepare material for publication: *SHELXL97*.

## Supplementary Material

Crystal structure: contains datablocks global, I. DOI: 10.1107/S1600536809020091/is2422sup1.cif

Structure factors: contains datablocks I. DOI: 10.1107/S1600536809020091/is2422Isup2.hkl

Additional supplementary materials:  crystallographic information; 3D view; checkCIF report

## Figures and Tables

**Table 1:** Hydrogen-bond geometry (Å, °)

*D*—H⋯*A*	*D*—H	H⋯*A*	*D*⋯*A*	*D*—H⋯*A*
C4—H4⋯*Cg*3^i^	0.93	2.88	3.7250 (3)	152
C13—H13⋯*Cg*3^ii^	0.93	2.94	3.5626 (3)	126
C18—H18*B*⋯*Cg*6^iii^	0.97	2.79	3.6726 (4)	152

Symmetry codes: (i) 
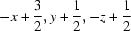
; (ii) 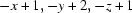; (iii) 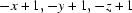. *Cg*3 and *Cg*6 are the centroids of the N2/C19–C22 and C11–C16 rings, respectively.

**Fig. 1.**
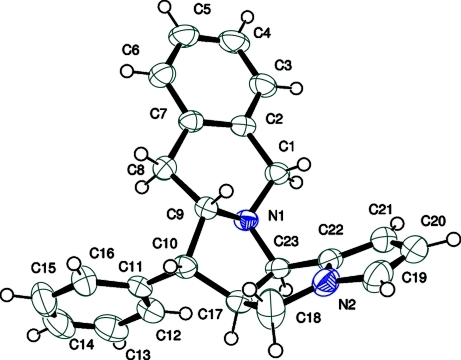
The molecular structure of (I), with atom labels and 50% probability displacement ellipsoids for non-H atoms.

## supplementary crystallographic information

### Comment

Pyrrolidine containing compounds are of significant importance because of their biological activities and widespread employment in catalysis (Witherup *et al.*, 1995; Kravchenko *et al.*, 2005). Pyrroles are very useful precursors in porphyrin synthesis (Sobral & Rocha Gonsalves, 2001*a*,*b*) and as monomers for polymer chemistry (Brockmann & Tour, 1995), with applications ranging from non linear optical materials (Suslick *et al.*, 1992) to electronic noses (Di Natale *et al.*, 1998).

The geometric parameters of the title molecule (Fig. 1) agree well with reported similar structure (Liu *et al.*, 2007). The phenyl ring (C11—C16) makes a dihedral angle of 83.43 (6)° with C2—C7 ring and 48.72 (6)° with N2/C19—C22 ring, respectively. The sum of the bond angles around N1 [334.71 (32)°] indicate the *sp*^3^ hybridized state of atom N1 in the molecule. The pyrrolidine ring [N1/C9/C10/C17/C23] adopts an envelope conformation.

The crystal structure is stabilized by weak C—H···π [C4—H4···*Cg*3, C13—H13···*Cg*3 & C18—H18B···*Cg*6 (Table 1; *Cg*3 and *Cg*6 are the centroid of the rings defined by the atoms N2/C19–C22 and C11–C16, respectively.)] interactions.

### Experimental

A mixture of *N*-allyl pyrrole-2-carbaldehyde (1 mmol) and 1,2,3,4-tetrahydroisoquinolinic acid (1 mmol) was refluxed in 1,2-dichloro benzene (10 ml) for 12 h till the completion of the reaction as evidenced by TLC analysis. The crude mixture was subjected to column chromatography to get the pure product.

### Refinement

H atoms were positioned geometrically and refined using riding model with C—H = 0.93 Å and *U*_iso_(H) = 1.2*U*_eq_(C) for aromatic C—H, C—H = 0.98 Å and *U*_iso_(H) = 1.2*U*_eq_(C) for C—H, and C—H = 0.97 Å and *U*_iso_(H) = 1.2*U*_eq_(C) for CH_2_,

### Crystal data

**Table:**

C_23_H_22_N_2_	*F*(000) = 696
*M**_r_* = 326.43	*D*_x_ = 1.268 Mg m^−^^3^
Monoclinic, *P*2_1_/*n*	Mo *K*α radiation, λ = 0.71073 Å
Hall symbol: -P 2yn	Cell parameters from 6264 reflections
*a* = 14.0694 (14) Å	θ = 2.0–28.4°
*b* = 5.9300 (5) Å	µ = 0.07 mm^−^^1^
*c* = 21.177 (2) Å	*T* = 293 K
β = 104.563 (3)°	Needle, yellow
*V* = 1710.1 (3) Å^3^	0.20 × 0.20 × 0.15 mm
*Z* = 4	

### Data collection

**Table:**

Bruker KappaAPEXII diffractometer	4266 independent reflections
Radiation source: fine-focus sealed tube	3049 reflections with *I* > 2σ(*I*)
graphite	*R*_int_ = 0.032
Detector resolution: 0 pixels mm^-1^	θ_max_ = 28.4°, θ_min_ = 2.0°
ω and φ scans	*h* = −18→17
Absorption correction: multi-scan (*SADABS*; Sheldrick, 1996)	*k* = −7→4
*T*_min_ = 0.985, *T*_max_ = 0.989	*l* = −28→28
20373 measured reflections	

### Refinement

**Table:**

Refinement on *F*^2^	Primary atom site location: structure-invariant direct methods
Least-squares matrix: full	Secondary atom site location: difference Fourier map
*R*[*F*^2^ > 2σ(*F*^2^)] = 0.049	Hydrogen site location: inferred from neighbouring sites
*wR*(*F*^2^) = 0.149	H-atom parameters constrained
*S* = 1.01	*w* = 1/[σ^2^(*F*_o_^2^) + (0.0757*P*)^2^ + 0.3733*P*] where *P* = (*F*_o_^2^ + 2*F*_c_^2^)/3
4266 reflections	(Δ/σ)_max_ < 0.001
226 parameters	Δρ_max_ = 0.17 e Å^−^^3^
0 restraints	Δρ_min_ = −0.23 e Å^−^^3^

### Special details

**Table:**

Geometry. All e.s.d.'s (except the e.s.d. in the dihedral angle between two l.s. planes) are estimated using the full covariance matrix. The cell e.s.d.'s are taken into account individually in the estimation of e.s.d.'s in distances, angles and torsion angles; correlations between e.s.d.'s in cell parameters are only used when they are defined by crystal symmetry. An approximate (isotropic) treatment of cell e.s.d.'s is used for estimating e.s.d.'s involving l.s. planes.
Refinement. Refinement of *F*^2^ against ALL reflections. The weighted *R*-factor *wR* and goodness of fit *S* are based on *F*^2^, conventional *R*-factors *R* are based on *F*, with *F* set to zero for negative *F*^2^. The threshold expression of *F*^2^ > σ(*F*^2^) is used only for calculating *R*-factors(gt) *etc*. and is not relevant to the choice of reflections for refinement. *R*-factors based on *F*^2^ are statistically about twice as large as those based on *F*, and *R*- factors based on ALL data will be even larger.

### Fractional atomic coordinates and isotropic or equivalent isotropic displacement parameters (Å^2^)

**Table:**

	*x*	*y*	*z*	*U*_iso_*/*U*_eq_	
N1	0.42095 (9)	0.02857 (19)	0.64155 (6)	0.0319 (3)	
N2	0.26180 (10)	0.2368 (2)	0.50434 (6)	0.0447 (3)	
C9	0.42973 (10)	0.2650 (2)	0.66188 (7)	0.0308 (3)	
H9	0.3639	0.3302	0.6548	0.037*	
C2	0.38439 (10)	−0.0647 (2)	0.74598 (7)	0.0353 (3)	
C8	0.48113 (11)	0.2811 (3)	0.73313 (7)	0.0380 (3)	
H8A	0.4760	0.4343	0.7480	0.046*	
H8B	0.5502	0.2470	0.7390	0.046*	
C10	0.48203 (10)	0.3722 (2)	0.61376 (7)	0.0347 (3)	
H10	0.4648	0.5326	0.6095	0.042*	
C1	0.35781 (11)	−0.0995 (2)	0.67344 (7)	0.0367 (3)	
H1A	0.3630	−0.2586	0.6642	0.044*	
H1B	0.2901	−0.0542	0.6556	0.044*	
C23	0.39150 (11)	0.0267 (2)	0.56953 (7)	0.0348 (3)	
H23	0.4220	−0.1011	0.5528	0.042*	
C17	0.43198 (12)	0.2536 (3)	0.54943 (8)	0.0400 (4)	
H17	0.4796	0.2248	0.5236	0.048*	
C7	0.43830 (10)	0.1217 (2)	0.77408 (7)	0.0357 (3)	
C11	0.59247 (11)	0.3523 (2)	0.63345 (7)	0.0375 (3)	
C6	0.45606 (12)	0.1552 (3)	0.84096 (8)	0.0476 (4)	
H6	0.4907	0.2820	0.8598	0.057*	
C22	0.28437 (11)	0.0379 (3)	0.53632 (7)	0.0392 (3)	
C3	0.35068 (12)	−0.2155 (3)	0.78562 (8)	0.0479 (4)	
H3	0.3141	−0.3403	0.7671	0.057*	
C12	0.64174 (12)	0.1645 (3)	0.61992 (8)	0.0472 (4)	
H12	0.6062	0.0414	0.5991	0.057*	
C14	0.79640 (14)	0.3355 (4)	0.66854 (11)	0.0724 (6)	
H14	0.8647	0.3305	0.6796	0.087*	
C5	0.42305 (13)	0.0033 (4)	0.87978 (8)	0.0555 (5)	
H5	0.4362	0.0266	0.9246	0.067*	
C16	0.64758 (13)	0.5309 (3)	0.66614 (9)	0.0531 (4)	
H16	0.6158	0.6585	0.6762	0.064*	
C4	0.37077 (14)	−0.1825 (4)	0.85217 (9)	0.0573 (5)	
H4	0.3489	−0.2863	0.8783	0.069*	
C21	0.19983 (13)	−0.0869 (3)	0.52436 (8)	0.0544 (5)	
H21	0.1929	−0.2310	0.5399	0.065*	
C20	0.12558 (14)	0.0442 (4)	0.48407 (9)	0.0637 (6)	
H20	0.0603	0.0017	0.4683	0.076*	
C19	0.16540 (13)	0.2435 (4)	0.47212 (9)	0.0579 (5)	
H19	0.1329	0.3616	0.4468	0.069*	
C18	0.34333 (14)	0.3849 (3)	0.50884 (10)	0.0663 (6)	
H18A	0.3341	0.5246	0.5304	0.080*	
H18B	0.3520	0.4195	0.4659	0.080*	
C13	0.74311 (14)	0.1569 (4)	0.63685 (10)	0.0634 (5)	
H13	0.7754	0.0302	0.6267	0.076*	
C15	0.74889 (15)	0.5212 (4)	0.68388 (11)	0.0696 (6)	
H15	0.7849	0.6409	0.7063	0.084*	

### Atomic displacement parameters (Å^2^)

**Table:**

	*U*^11^	*U*^22^	*U*^33^	*U*^12^	*U*^13^	*U*^23^
N1	0.0392 (6)	0.0291 (6)	0.0299 (6)	−0.0006 (5)	0.0136 (5)	0.0012 (4)
N2	0.0475 (8)	0.0488 (8)	0.0345 (7)	−0.0014 (6)	0.0043 (6)	0.0001 (6)
C9	0.0322 (7)	0.0279 (6)	0.0335 (8)	0.0011 (5)	0.0103 (6)	0.0010 (5)
C2	0.0359 (7)	0.0397 (7)	0.0328 (8)	0.0054 (6)	0.0133 (6)	0.0054 (6)
C8	0.0399 (8)	0.0399 (8)	0.0338 (8)	−0.0039 (6)	0.0085 (6)	−0.0013 (6)
C10	0.0406 (8)	0.0294 (7)	0.0356 (8)	−0.0011 (5)	0.0127 (6)	0.0035 (5)
C1	0.0450 (8)	0.0321 (7)	0.0358 (8)	−0.0041 (6)	0.0157 (6)	0.0006 (6)
C23	0.0443 (8)	0.0327 (7)	0.0314 (7)	−0.0013 (6)	0.0167 (6)	−0.0013 (5)
C17	0.0456 (8)	0.0423 (8)	0.0343 (8)	−0.0065 (6)	0.0143 (7)	0.0043 (6)
C7	0.0325 (7)	0.0436 (8)	0.0317 (8)	0.0064 (6)	0.0095 (6)	0.0033 (6)
C11	0.0399 (8)	0.0385 (8)	0.0373 (8)	−0.0059 (6)	0.0158 (6)	0.0057 (6)
C6	0.0465 (9)	0.0617 (10)	0.0339 (8)	−0.0002 (7)	0.0086 (7)	−0.0013 (7)
C22	0.0477 (9)	0.0414 (8)	0.0305 (7)	−0.0066 (6)	0.0132 (6)	−0.0036 (6)
C3	0.0492 (9)	0.0544 (10)	0.0439 (9)	−0.0047 (7)	0.0187 (8)	0.0083 (7)
C12	0.0464 (9)	0.0467 (9)	0.0530 (10)	−0.0005 (7)	0.0210 (8)	0.0029 (7)
C14	0.0384 (10)	0.1029 (18)	0.0768 (15)	−0.0080 (11)	0.0159 (10)	0.0205 (13)
C5	0.0539 (10)	0.0837 (13)	0.0305 (8)	0.0026 (9)	0.0138 (7)	0.0060 (8)
C16	0.0524 (10)	0.0499 (10)	0.0568 (11)	−0.0141 (8)	0.0136 (8)	−0.0027 (8)
C4	0.0572 (11)	0.0792 (13)	0.0411 (10)	−0.0012 (9)	0.0227 (8)	0.0172 (9)
C21	0.0581 (11)	0.0679 (12)	0.0391 (9)	−0.0228 (9)	0.0157 (8)	−0.0059 (8)
C20	0.0449 (10)	0.1055 (17)	0.0405 (10)	−0.0142 (10)	0.0105 (8)	−0.0098 (10)
C19	0.0474 (10)	0.0791 (13)	0.0413 (10)	0.0060 (9)	0.0006 (8)	−0.0050 (9)
C18	0.0655 (12)	0.0555 (11)	0.0623 (12)	−0.0168 (9)	−0.0126 (10)	0.0245 (9)
C13	0.0515 (11)	0.0744 (13)	0.0722 (14)	0.0120 (9)	0.0303 (10)	0.0125 (10)
C15	0.0526 (11)	0.0799 (14)	0.0719 (14)	−0.0276 (11)	0.0074 (10)	0.0018 (11)

### Geometric parameters (Å, °)

**Table:**

N1—C1	1.4574 (17)	C11—C12	1.380 (2)
N1—C9	1.4625 (17)	C11—C16	1.390 (2)
N1—C23	1.4764 (18)	C6—C5	1.377 (2)
N2—C19	1.357 (2)	C6—H6	0.9300
N2—C22	1.358 (2)	C22—C21	1.369 (2)
N2—C18	1.429 (2)	C3—C4	1.380 (2)
C9—C8	1.503 (2)	C3—H3	0.9300
C9—C10	1.5366 (19)	C12—C13	1.381 (2)
C9—H9	0.9800	C12—H12	0.9300
C2—C7	1.387 (2)	C14—C15	1.369 (3)
C2—C3	1.389 (2)	C14—C13	1.372 (3)
C2—C1	1.501 (2)	C14—H14	0.9300
C8—C7	1.507 (2)	C5—C4	1.371 (3)
C8—H8A	0.9700	C5—H5	0.9300
C8—H8B	0.9700	C16—C15	1.381 (3)
C10—C11	1.509 (2)	C16—H16	0.9300
C10—C17	1.537 (2)	C4—H4	0.9300
C10—H10	0.9800	C21—C20	1.405 (3)
C1—H1A	0.9700	C21—H21	0.9300
C1—H1B	0.9700	C20—C19	1.359 (3)
C23—C22	1.496 (2)	C20—H20	0.9300
C23—C17	1.561 (2)	C19—H19	0.9300
C23—H23	0.9800	C18—H18A	0.9700
C17—C18	1.537 (2)	C18—H18B	0.9700
C17—H17	0.9800	C13—H13	0.9300
C7—C6	1.389 (2)	C15—H15	0.9300
			
C1—N1—C9	112.29 (11)	C12—C11—C16	118.18 (15)
C1—N1—C23	115.43 (11)	C12—C11—C10	122.81 (14)
C9—N1—C23	106.99 (10)	C16—C11—C10	119.01 (14)
C19—N2—C22	110.79 (15)	C5—C6—C7	120.94 (17)
C19—N2—C18	134.42 (16)	C5—C6—H6	119.5
C22—N2—C18	114.69 (14)	C7—C6—H6	119.5
N1—C9—C8	109.89 (11)	N2—C22—C21	107.09 (15)
N1—C9—C10	102.73 (11)	N2—C22—C23	110.77 (13)
C8—C9—C10	116.72 (12)	C21—C22—C23	142.08 (16)
N1—C9—H9	109.1	C4—C3—C2	120.85 (17)
C8—C9—H9	109.1	C4—C3—H3	119.6
C10—C9—H9	109.1	C2—C3—H3	119.6
C7—C2—C3	119.11 (14)	C11—C12—C13	120.81 (17)
C7—C2—C1	121.14 (12)	C11—C12—H12	119.6
C3—C2—C1	119.67 (14)	C13—C12—H12	119.6
C9—C8—C7	112.12 (12)	C15—C14—C13	119.86 (18)
C9—C8—H8A	109.2	C15—C14—H14	120.1
C7—C8—H8A	109.2	C13—C14—H14	120.1
C9—C8—H8B	109.2	C4—C5—C6	119.76 (16)
C7—C8—H8B	109.2	C4—C5—H5	120.1
H8A—C8—H8B	107.9	C6—C5—H5	120.1
C11—C10—C9	114.59 (11)	C15—C16—C11	120.79 (19)
C11—C10—C17	114.82 (12)	C15—C16—H16	119.6
C9—C10—C17	102.09 (11)	C11—C16—H16	119.6
C11—C10—H10	108.3	C5—C4—C3	119.97 (16)
C9—C10—H10	108.3	C5—C4—H4	120.0
C17—C10—H10	108.3	C3—C4—H4	120.0
N1—C1—C2	112.25 (12)	C22—C21—C20	106.99 (17)
N1—C1—H1A	109.2	C22—C21—H21	126.5
C2—C1—H1A	109.2	C20—C21—H21	126.5
N1—C1—H1B	109.2	C19—C20—C21	108.40 (17)
C2—C1—H1B	109.2	C19—C20—H20	125.8
H1A—C1—H1B	107.9	C21—C20—H20	125.8
N1—C23—C22	118.24 (11)	N2—C19—C20	106.73 (17)
N1—C23—C17	104.38 (11)	N2—C19—H19	126.6
C22—C23—C17	103.13 (12)	C20—C19—H19	126.6
N1—C23—H23	110.2	N2—C18—C17	104.54 (13)
C22—C23—H23	110.2	N2—C18—H18A	110.8
C17—C23—H23	110.2	C17—C18—H18A	110.8
C18—C17—C10	112.99 (14)	N2—C18—H18B	110.8
C18—C17—C23	106.78 (13)	C17—C18—H18B	110.8
C10—C17—C23	105.64 (11)	H18A—C18—H18B	108.9
C18—C17—H17	110.4	C14—C13—C12	120.24 (19)
C10—C17—H17	110.4	C14—C13—H13	119.9
C23—C17—H17	110.4	C12—C13—H13	119.9
C2—C7—C6	119.35 (14)	C14—C15—C16	120.1 (2)
C2—C7—C8	120.50 (13)	C14—C15—H15	120.0
C6—C7—C8	120.09 (14)	C16—C15—H15	120.0
			
C1—N1—C9—C8	65.15 (15)	C17—C10—C11—C16	146.25 (14)
C23—N1—C9—C8	−167.22 (11)	C2—C7—C6—C5	1.6 (2)
C1—N1—C9—C10	−169.97 (11)	C8—C7—C6—C5	−175.69 (15)
C23—N1—C9—C10	−42.33 (13)	C19—N2—C22—C21	−0.11 (19)
N1—C9—C8—C7	−48.57 (15)	C18—N2—C22—C21	−177.00 (16)
C10—C9—C8—C7	−164.97 (12)	C19—N2—C22—C23	177.62 (13)
N1—C9—C10—C11	−84.82 (14)	C18—N2—C22—C23	0.7 (2)
C8—C9—C10—C11	35.46 (17)	N1—C23—C22—N2	112.17 (14)
N1—C9—C10—C17	39.94 (13)	C17—C23—C22—N2	−2.34 (15)
C8—C9—C10—C17	160.22 (12)	N1—C23—C22—C21	−71.4 (3)
C9—N1—C1—C2	−49.38 (16)	C17—C23—C22—C21	174.1 (2)
C23—N1—C1—C2	−172.38 (11)	C7—C2—C3—C4	−0.4 (2)
C7—C2—C1—N1	20.18 (19)	C1—C2—C3—C4	−177.42 (15)
C3—C2—C1—N1	−162.92 (13)	C16—C11—C12—C13	−1.7 (2)
C1—N1—C23—C22	38.60 (17)	C10—C11—C12—C13	177.70 (15)
C9—N1—C23—C22	−87.17 (14)	C7—C6—C5—C4	−0.8 (3)
C1—N1—C23—C17	152.43 (12)	C12—C11—C16—C15	0.7 (3)
C9—N1—C23—C17	26.66 (14)	C10—C11—C16—C15	−178.73 (16)
C11—C10—C17—C18	−142.72 (14)	C6—C5—C4—C3	−0.6 (3)
C9—C10—C17—C18	92.68 (14)	C2—C3—C4—C5	1.3 (3)
C11—C10—C17—C23	100.90 (14)	N2—C22—C21—C20	−0.04 (19)
C9—C10—C17—C23	−23.71 (14)	C23—C22—C21—C20	−176.58 (19)
N1—C23—C17—C18	−121.16 (14)	C22—C21—C20—C19	0.2 (2)
C22—C23—C17—C18	3.00 (16)	C22—N2—C19—C20	0.2 (2)
N1—C23—C17—C10	−0.62 (14)	C18—N2—C19—C20	176.3 (2)
C22—C23—C17—C10	123.53 (12)	C21—C20—C19—N2	−0.2 (2)
C3—C2—C7—C6	−1.0 (2)	C19—N2—C18—C17	−174.65 (17)
C1—C2—C7—C6	175.95 (13)	C22—N2—C18—C17	1.3 (2)
C3—C2—C7—C8	176.34 (14)	C10—C17—C18—N2	−118.36 (16)
C1—C2—C7—C8	−6.7 (2)	C23—C17—C18—N2	−2.7 (2)
C9—C8—C7—C2	20.92 (19)	C15—C14—C13—C12	0.5 (3)
C9—C8—C7—C6	−161.79 (13)	C11—C12—C13—C14	1.2 (3)
C9—C10—C11—C12	84.56 (17)	C13—C14—C15—C16	−1.5 (3)
C17—C10—C11—C12	−33.18 (19)	C11—C16—C15—C14	0.9 (3)
C9—C10—C11—C16	−96.00 (16)		

### Hydrogen-bond geometry (Å, °)

**Table:**

*D*—H···*A*	*D*—H	H···*A*	*D*···*A*	*D*—H···*A*
C4—H4···Cg3^i^	0.93	2.88	3.7250 (3)	152
C13—H13···Cg3^ii^	0.93	2.94	3.5626 (3)	126
C18—H18B···Cg6^iii^	0.97	2.79	3.6726 (4)	152

Symmetry codes: (i) −*x*+3/2, *y*+1/2, −*z*+1/2; (ii) −*x*+1, −*y*+2, −*z*+1; (iii) −*x*+1, −*y*+1, −*z*+1.
